# Physiotherapy interventions in post- and long-COVID-19: a scoping review of the literature up to February 2023

**DOI:** 10.1186/s12913-025-13631-7

**Published:** 2025-10-30

**Authors:** Judith Gartmann, Christian Sturm, Andrea Bökel

**Affiliations:** Department of Rehabilitation und Sports Medicine, Hannover Medical School, Carl-Neuberg-Straße 1, 30625 Hannover, OE 4250 Germany

**Keywords:** Physical therapy modalities, Post-acute COVID-19 syndrome, Scoping review, Treatment parameter, Treatment outcome

## Abstract

**Background:**

Physiotherapy is an accepted and recommended treatment for post- and long-COVID-19 condition. It is assumed that physiotherapists reported a lack of information on physiotherapy interventions and treatment parameters. This scoping review provides an overview of the evidence on physiotherapy interventions published in the scientific literature.

**Methods:**

The scoping review includes studies analysing physiotherapy treatment parameters and interventions in post- and long-COVID patients. Studies focusing on telemedicine or exclusively home-based workouts are excluded. A systematic literature search was conducted by two independent researchers in the databases PubMed, EBSCO, SCOPUS, Web of Science, EMBASE, PEDro, Cochrane, and WISO. Following the abstract screening process, data extraction and critical assessment were performed.

**Results:**

In this scoping review, 6 studies were included, providing information physiotherapy management of post- and long-COVID-19. The research identified respiratory therapy, aerobic training or strength training as key areas of focus.

**Conclusion:**

This scoping review highlights the need for further studies on physiotherapy treatment in post- and long-COVID condition.

**Trial registration:**

Open Science Framework (Registry number osf.io/5k3tA).

**Supplementary Information:**

The online version contains supplementary material available at 10.1186/s12913-025-13631-7.

## Background

The prevalence of long-COVID varies across different studies, ranging from 12.7 and 43% depending on the source [1–3]. Various systematic reviews and meta-analyses have concluded that long-COVID is more prevalent in hospitalised adults with severe cases of COVID-19 than in those with milder cases that do not require hospitalisation [3, 4]. Patients with long-COVID can exhibit a wide range of symptoms. The most common symptoms are fatigue (47%), dyspnoea (32%), myalgia (25%), joint pain (20%), headache (18%), cough (18%), and chest pain (15%) [5]. Due to this wide variety of symptoms and the numerous potential comorbidities, many authors recommend a multimodal, multidisciplinary and individually tailored rehabilitation approach [6, 7] which also includes physiotherapy and symptom self-management. Treatment is symptom-oriented [8, 9]. As physiotherapy interventions show positive effects in the treatment of symptoms of chronic obstructive pulmonary disease [10–12], fibromyalgia [13] or HIV [14, 15] it is a worldwide accepted and recommended treatment in rehabilitation.

Physiotherapists working in outpatient practices are uncertain about how to treat the long-term effects of the COVID-19 virus [16].

A preliminary search on physiotherapy interventions was conducted in 2021 using the following databases: PubMed, Pedro, Embase and Cochrane Database. The search revealed no current or ongoing systematic or scoping reviews on the topic of physiotherapy treatment parameters. Nevertheless, the preliminary search demonstrated that multidisciplinary rehabilitation in infectious diseases and physiotherapy is a recommended treatment for post- and long-COVID symptoms, including pulmonary symptoms and pain [17–24].

Systematic reviews conclude that rehabilitation using an exercise programme could improve both physical and psychological outcomes [25, 26] as well as quality of life [27]. However, the relevant training guidelines regarding the frequency, intensity, timing and type of exercise for long-term rehabilitation are not yet fully clear [25, 26]. Therefore, high-quality clinical studies need to be developed and implemented [18, 22, 28–31]. Due to the lack of studies to date, rehabilitation is partly based on expert opinion [32] and on research into rehabilitation methods proven to be effective in treating similar symptoms in different contexts [16, 33].

This scoping review aimed to summarise the available evidence on physiotherapy interventions and treatment parameters for managing patients with post- and long-COVID conditions.

## Methods

This scoping review was registered in on Open Science Framework (Registry number http://osf.io/5k3tA). As no patients and public were involved a positive vote of an ethical committee was not necessary.

The proposed scoping review was conducted in accordance with the Joanna Briggs Institute (JBI) methodology for scoping reviews [34, 35]. The Preferred Reporting Items for Systematic Reviews and Meta-Analyses (PRISMA) checklist extension for scoping reviews was used for reporting [36].

The methodological framework provided by the JBI was used. This methodological framework includes several steps from defining the research question and the eligibility criteria, conducting the literature research, study selection and data extraction. Additionally, a critical appraisal was conducted using the JBI critical appraisal tools. The quality of the methodology of the selected studies was evaluated with regard to the extracted data.

### Review question

The primary question guiding this scoping review is ‘What kind of physiotherapeutic interventions are used in both outpatient and inpatient settings for post- and long-COVID-19 patients?’ This question is divided into subquestions, as illustrated in Table 1.

**Table 1:** Sub-questions of the scoping review

Perspective	Research questions
(1) Descriptive	a) How many studies were published between January 2020 and February 2023 on physiotherapy interventions in patients with post- or long-COVID-Syndrome?
(2) Methods	a) Which research methods were used in the published literature? b) What population characteristics were described in the published literature? c) What definitions of post- and long-COVID-19 were used in the published literature?
(3) Physiotherapy interventions	a) What types of physiotherapy interventions were reported in the published literature? b) What was the reported frequency of the physiotherapeutic interventions? c) What was the reported intensity of the physiotherapeutic interventions?
(4) Physiotherapy and Post-/Long-COVID	a) What was the impact of physiotherapy on post- or long-COVID-19 symptoms? b) Were adverse events reported for physiotherapeutic interventions in patients with post- or long- COVID syndrome? c) Were negative influences reported for parallel, applied physiotherapeutic interventions in patients with post- or long-COVID syndrome? d) Were differences reported in- and outpatient interventions?

### Eligibility criteria

The decision to include only German and English language studies in this study was based on the authors’ proficiency in both, thus allowing for a more accurate analysis and interpretation of the data. Studies addressing post-COVID, post-acute COVID and long- COVID were included. Studies that also examined acute COVID-19 or COVID-19 in general were only considered eligible if the results pertaining to post- COVID, post-acute COVID or long- COVID were reported separately and in a comprehensible format. Randomised controlled trials, observational studies, case reports, umbrella reviews, rapid reviews focusing on post- and long-COVID-19 in adults were considered for inclusion in this scoping review, when published between January 2020 and February 2023. Qualitative studies were excluded due to the quantitative nature of the analysis in this study, which focuses on treatment details. Grey literature, text and opinion papers were not considered for inclusion in this scoping review.

Studies analysing telemedicine and solely home-based workouts were not included in this study because the telemedicine approach has not yet been widely established in German physiotherapy care.

Eligibility criteria are shown in Table 2.

**Table 2:** Eligibility criteria

Inclusion criteria	Exclusion criteria
Long-COVID-19 Post-COVID-19 Post-acute COVID Adult patients aged above 18 years Physiotherapy interventions Physiotherapy interventions with additional home exercise workout Randomised controlled trials, observational studies, case reports, umbrella reviews, rapid reviews German or English language Publication date between January 2020 and February 2023	Acute COVID-19 Non-COVID-19 Other infectious diseases Other respiratory disorders Non-systematic reviews Study protocols Editorials, letters Narrative reviews Qualitative studies Grey literature Children and adolescent patients aged under 18 years Telemedicine Solely home-based workouts

### Sources

Databases PubMed, EBSCO, Scopus, Web of Science, Embase, PEDRO, Cochrane and WISO were searched by two independent researchers. A professional librarian was not involved.

### Search strategy

The search strategy is focused on published studies. The primary search strategy is based on the 4 domains of population, physiotherapy intervention, outcome, study design shown in Table 3. The secondary search strategy involves the combination of these domains: ‘population AND physiotherapy intervention’, ‘population AND physiotherapy intervention AND outcome’, and ‘population AND physiotherapy intervention AND outcome AND study design’.

**Table 3:** Search terms and MeSH

Domain	Search terms	MeSH terms
Population	Long-COVID, post-COVID-19, Post-acute COVID	”Post-acute COVID-19 syndrome”
Physiotherapy intervention	Physiotherapy, aerobic exercise, physical activity, resistance training, passive mobilisation, active mobilization, manual therapy, massage therapy, lymphatic drainage, electrotherapy, ultrasound, respiratory therapy, proprioceptive neuromuscular facilitation (PNF), Vojta, Bobath, pressure ulcer management, prevention	“Physical therapy modalities“OR “Physical therapy specialty“OR”Exercise therapy” OR “Exercise movement techniques” OR “Physical fitness“OR “Musculoskeletal manipulation” OR “Relaxation” OR “Stress, physiological” OR “Immobilisation” OR “Prevention & control” OR “Manual lymphatic drainage“OR “Electric stimulation therapy“OR “Ultrasonic therapy“OR “Respiratory therapy“OR “Breathing exercises“
Outcome	Physical fitness, fatigue, general health, pain, return to work, physical health, mental health, mobility, vital capacity, dyspnoea, myalgia, arthralgia, functional capacity, health-related quality of life, stress, range of movement, patient-reported outcome measures	“Physical fitness“OR “Fatigue“OR “Mental health” OR “Return to work“OR “Pain management” OR “Vital capacity“OR “Myalgia“OR “Arthralgia” OR “Quality of life“OR “Patient-reported outcome measures“OR “Health” OR “Health status”
Study design	Randomised control trial Observational studies Case reports	“Randomised controlled trial” OR “Observational study” OR “Case reports”

The search terms and relevant keywords were identified and deposited with medical subject headings (MeSH) (Table 3). The strategies for the literature search are listed in the Appendix I.

### Screening process

Two independent researchers collected the identified literature in separate Excel sheets and removed duplicates. Titles and abstracts were then screened by two independent reviewers to ascertain whether they met the inclusion criteria for the scoping review. Thereafter, the full-text articles that met the inclusion criteria in the title and abstract screening process were screened independently by the two researchers with the aim of identifying the inclusion criteria. The reasons for the exclusion of sources of evidence at full-text that do not meet the inclusion criteria were recorded and reported in the scoping review. In the next step both independent researchers compared their search results. Any disagreement was resolved through discussion or consulting with a third independent researcher.

### Critical appraisal

Additionally, a critical appraisal of each included article was conducted by the two independent researchers using the JBI critical appraisal tools [37]. The quality assessment evaluated the methodological quality with regard to the extracted data on physiotherapy interventions and treatment parameters. Thereafter, both researchers compared the results of the critical appraisal. In the event of differences in the quality report the results were discussed or a third independent researcher was involved for reaching a consensus.

### Data extraction

Data was extracted from the included papers by two reviewers independently using a data extraction tool that has been developed by the reviewers. The data extraction tool was created in MS Word providing specific details on the study design, research method, population characteristics, definition of post- and long- COVID-19, intervention and key findings relevant to the review question/s, e. g. these included frequency and intensity of the intervention, the outcome of the study and adverse events.

## Results

The literature search was conducted in March 2023.

### Descriptive

#### How many studies were published between January 2020 and February 2023 on physiotherapy interventions in patients with post- or long-COVID syndrome?

**Fig. 1:**
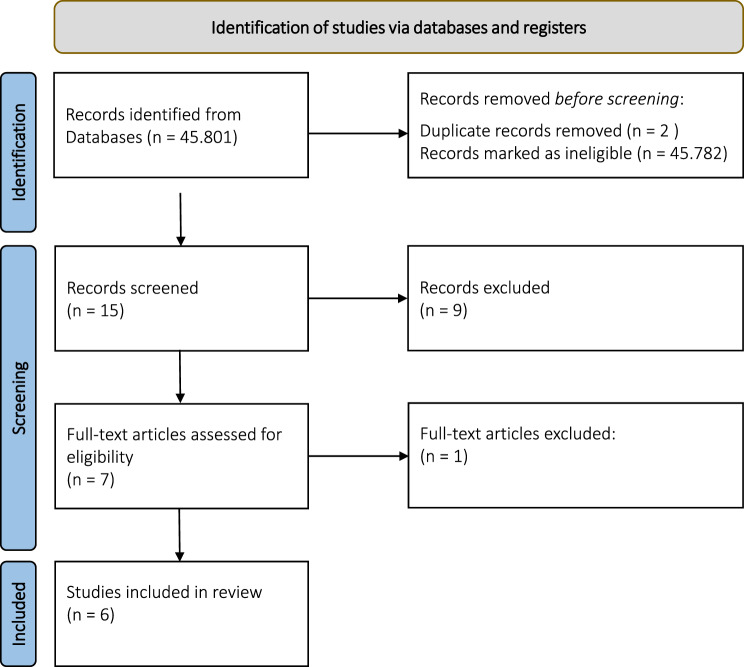
Preferred reporting items for systematic reviews and meta-analysis (PRISMA) flow diagram [36]

45.801 articles were screened on the inclusion criteria. 19 papers remained. After reduction of duplets 15 articles remained for the title and abstract screening process after which 9 studies were excluded. Finally, 6 studies were included in the scoping review (Table 4). A flow diagram can be found in Fig. 1 [38].

**Table 4:** Illustration of the included studies

No.	Author	Year	Country	Study design	Objective
**Post-COVID-19**					
1	Ali AA et al. [20] DOI: 10.47750/pnr.2023.14.S02.87.	2023	Egypt	Randomized controlled trial, prospective, single-blinded, two groups, before and post-test analysis,	’to determine how active cycle breathing affects specific pulmonary outcomes in patients having post-COVID syndrome.’ [20]
2	Nagy EN et al. [39] DOI: 10.2340/jrm.v54.3972.	2022	Egypt	Randomized controlled trial, Study group and control group	’to evaluate the combined effect of manual DR and IMT compared with IMT alone on selected parameters (blood pressure, dyspnoea, high serum lactate, and fatigue levels) in men with post-acute sequelae of COVID-19 syndrome.’ [39]
3	Nambi G et al. [19] DOI: 10.1177/026921552110369	2022	Saudi Arabia,	Randomized, single-blinded, Low intensity aerobic training group (LAT) versus high intensity aerobic training group (HAT) prospective, clinical study	’to find and compare the clinical and psychological effects of low and high-intensity aerobic training combined with resistance training in community-dwelling older men with post-COVID-19 sarcopenia symptoms.’ [19]
4	Rutkowski S et al. [40] DOI: 10.3389/fpubh.2023.1121554.	2023	Poland, United States of America	Randomized controlled trial; VR group versus control group both with 4 pulmonary rehabilitation models	’to propose an innovative comprehensive intervention based on a pulmonary rehabilitation programme for patients with post-acute sequelae of COVID-19.’ [40]
5	Mayer KP et al. [21] DOI: 10.1093/ptj/pzab098.	2021	United States of America	Case Report	’to present the clinical presentation and physical therapist management for a patient with post–COVID syndrome.’ [21]
**SARS-CoV-2: post-COVID-19**					
6	Santos S et al. [24] DOI: 10.1002/pri.1938.	2022	Peru	Case Report	describe ‘an intervention based on musculoskeletal physiotherapy in a post-COVID-19 adult woman with physical sequelae’. [24]

### Methods

#### Which research methods were used in the published literature?

The majority of the studies were published in 2022 (*n* = 5) concentrating on ‘post-COVID-19’. No published study focusing on ‘long-COVID-19’was identified. One study used ‘SARS-CoV-2’ and defined the subtype of ‘post-COVID-19’ in the abstracts [24]. Two-thirds of the 6 included studies were randomized controlled trials (*n* = 4) [19, 20, 39, 40].

#### What population characteristics were described in the published literature?

The published population characteristics are shown in Table 5.

**Table 5:** Population characteristics of the included studies

No.	Author	Population	Healthcare delivery setting
**Post-COVID-19**			
1	Ali AA et al. [20]	60 patients both sexes (31 female, 29 male), with difficulty in breathing during activities of daily living (dyspnoea, cough and sputum production) Age in years: 45.62 ± 2.47 versus 45.7 ± 2.33	N. a.
2	Nagy EN et al. [39]	52 men with post-COVID-19 syndrome, Age in years: 40.00 ± 3.36 versus 39.70 ± 3.55	Outpatient
3	Nambi G et al. [19]	65 men, post COVID 19 sarcopenia Age in years: 63.2 ± 3.1 versus 64.1 ± 3.2	N. a.
4	Rutkowski S et al. [40]	32 patients (20 female, 12 male) with a confirmation from a primary care physician of having had COVID-19 infection, Age in years: 57,8 ± 4,92	Inpatient
5	Mayer KP et al.[21]	Woman 37 years old, day 62 after initial infection	Outpatient
**SARS-CoV-2: post-COVID-19**			
6	Santos S et al.[24]	Woman, 60 years old, 28 days after initial infection	Outpatient (home visit)

#### What definitions of post- and long-COVID-19 were used in the published literature?

Hardly any definitions for post- and long-COVID-19 were identified. The authors of the included literature referred to other authors and described the possible symptoms of this post infectious condition (Table 6).

**Table 6:** Definitions of post- and long-COVID-19 used in the included studies

No.	Author	Definition post- and long-COVID-19
**Post-COVID-19**		
1	Ali AA et al. [20]	’ … PCS is a condition in which signs and symptoms persist for more than 4 weeks following an acute infection.’ [41]
2	Nagy EN et al. [39]	No definition identified
3	Nambi G et al. [19]	No definition identified
4	Rutkowski et al. [40]	No definition identified
5	Mayer KP et al. [21]	’two definitions of post-acute COVID-19 are given: (1) ongoing symptomatic COVID-19 for people who still have symptoms between 4 and 12 weeks after the start of acute symptoms; and (2) post-COVID-19 syndrome for people who still have symptoms for more than 12 weeks after the start of acute symptoms.’ [42]
**SARS-CoV-2: post-COVID-19**		
6	Santos S et al. [24]	No definition identified

### Physiotherapy interventions

#### What types of physiotherapy interventions were reported in the published literature?

The research of physiotherapy management of post- and long-COVID-19 identified on the following key areas of focus: respiratory therapy [20, 21, 39, 40], aerobic training [19–21, 40] or strength training [19–21, 40]. One study reported ‘hands-on’ physiotherapy interventions like manual therapy and TENS [24]. The interventions are reported in Table 7.

**Table 7:** Reported physiotherapy interventions and treatment parameters

Category	Interventions	Frequency and duration in therapy	Intensity	Frequency in general	Source
Respiratory therapy	Post-COVID-19				
	- Respiratory exercise with active cycle of breathing technique, controlled breathing, - thoracic expansion exercises, forced expiration technique, - active diaphragma release techniques, - pursed-lip abdominal breathing exercise	- 2-3 times: 20–30 seconds controlled breathing, Thoracic expansion exercises 3–4 times, normal breath 20–30 seconds or 6 breaths - daily 3–4 times maximum of 3 min, 10 breaths, 20 seconds rest - 4-6 seconds		1-3 sessions per week for 12 weeks	Ali AA et al. [20]
	- Diaphragm release - Inspiration muscle Training with tool	2x 10 breaths, 1 minute rest 4 min, twice a day 30 breaths, 2 min rest		3 sessions per week for 6 weeks	Nagy EN et al. [39]
	- High-intensity pulmonary programme with breathing exercise			5 sessions per week for 3 weeks	Rutkowski et al. [40]
	Diaphragm breathing techniques			15 sessions in 8 weeks	Mayer KP et al. [21]
Aerobic training	Post-COVID-19				
	- Treadmill	20 min	Increased each day regarding to patients’ tolerability	3 sessions per week for 12 weeks	Ali AA et al. [20]
	- Treadmill - Cycle ergometer	20 min 10 min	Low-intensity aerobic training (40-60% of maximum heart rate) or high-intensity aerobic training (60-80% of maximum heart peak)	4 sessions per week for 8 weeks	Nambi G et al. [19]
	- Cycle ergometer with or without Virtual Reality	Twice a day	Heart rate increase of 90%/80%/70%/20-30% 50-70 RPM	5 sessions per week for 3 weeks	Rutkowski et al. [40]
	- Crosstrainer, treadmill, jogging, upper extremity bicycle ergometer - Dancing	15-45 min 3-6 min	- Initial level 60-80% Heart rate max, Increased to mRPE scale 4/10 (somewhat hard) to 6/10 (hard) - cadence 80 bpm	15 sessions in 8 weeks	Mayer KP et al. [21]
Strength training	Post-COVID-19				
	- Active limb exercises with weight machines, free weights, elastic bands	10 - 45 min 3 sets of 8–12 repetition with progression to 15 repetitions, with 1 min rest		1-3 sessions per week for 12 weeks	Ali AA et al. [20]
	- With weights	3 times 10 repetitions, 1 min rest		4 sessions per week for 8 weeks	Nambi G et al. [19]
	- General fitness exercises, resistance training and relaxation			5 sessions per week for 3 weeks	Rutkowski et al. [40]
	- Weight machines, dumbbells, ankle weights, Core stability - Home workout with elastic bands	10-20 min 3 times per week 3x15 repetitions		15 sessions in 8 weeks	Mayer KP et al. [21]
‘Hands-on’ physiotherapy	Post-COVID-19				
	- Stretching for warm- up and cool-down				Nambi G et al. [19]
	SARS-CoV-2: post-acute COVID-19				
	- Stretching	From 4^th^ session on		15 sessions in 5 weeks; 1:15 h - 1:50 h	Santos S et al. [24]
	- Transcutaneous electric nerve stimulation	Analgesic type, tetrapolar, 20 min	60-100Hz	15 sessions in 5 weeks; 1:15 h - 1:50 h	Santos S et al. [24]
	- Cyriax Deep Transverse Friction Massage bilateral brachial biceps and supraspinosus muscle			15 sessions in 5 weeks; 1:15 h - 1:50 h	Santos S et al. [24]
	- kinesitherapy	4^th^ to 5^th^ 6^th^ to 10^th^ 11^th^ to 15th	Passive Active body segment weight, increasing with 1/2 kg, 1 kg and up to 2 Kg	15 sessions in 5 weeks; 1:15 h - 1:50 h	Santos S et al. [24]
	- Maitlands’ manual therapy spine	6^th^ to 10^th^ session		15 sessions in 5 weeks; 1:15 h - 1:50 h	Santos S et al. [24]

#### What was the reported frequency of the physiotherapeutic interventions?

The frequency varied from 3 to 12 weeks, with 1 to 5 sessions weekly, respectively [19–21, 24, 39, 40]. It is shown in Table 7.

#### What was the reported intensity of the physiotherapeutic interventions?

The intensity was not reported in every intervention. The intensity practiced found in the included literature is shown in Table 7 [20, 21, 24, 40].

### Physiotherapy and post- and/or long-COVID-19

#### What was the impact of physiotherapy on post- or long-COVID symptoms?

Overall physiotherapy resulted in a change in health status parameters, including a reduction in fatigue levels, an improvement in respiratory function, and an enhancement in physical capacity. Arterial oxygen saturation and partial pressure of oxygen increased [20]. However, muscle mass remained unaltered [19]. The results are shown in Table 8.

**Table 8:** Impact of physiotherapy on health status parameters

Health status parameters	Outcome	Outcome scores or scales	Statistics	Source
Fatigue	↓	Fatigue Assessment Scale	Mean difference (% of change); p-value Group A 14.03 (35.92); 0.001 Group B 23.6 (61.05); 0.001	Ali AA et al. [20]
		Fatigue Severity Scale	Mean difference (SD); p-value Study group from 43.36 (±5.25) to 28.68 (±6.01); < 0.001 control group from 42.47 (±5.18) to 39.77 (±5.89); 0.001	Nagy EN et al. [39]
		Borg scale while 6-minute walk test	Group Mean (median); p-value VR group from 3.0 (1.0) to 2.0 (1.0); 0.055 Control group from 3.0 (1.0) to 2.0 (1.0); 0.010	Rutkowski et al. [40]
	↑	Subjective description	N. a.	Mayer KP et al. [21]
Walking distance	↑	6-minute walk test	Mean difference (% of change); p-value Group A − 53.03 (11.09); 0.001 Group B − 105.27 (21.61); 0.001	Ali AA et al. [20]
			Mean difference (SD); p-value Study group 56.50 m 13.53%; < 0.001 Control group from 16.50 m 3.92%; < 0.001	Nagy EN et al. [39]
			Group Mean ± SD in m; p-value VR group from 502 ± 48.8 to 558 ± 76; 0.010 Control group from 512 ± 54.3 to 552 ± 49.1; < 0.001	Rutkowski et al. [40]
			Difference: from 312 m to 511 m (+199 m)	Mayer KP et al. [21]
Arterial oxygen saturation	↑	Arterial blood gas analysis	Mean difference (% of change); p-value Group A − 2.4 (2.70); 0.001 Group B − 5.7 (6.43); 0.001	Ali AA et al. [20]
partial pressure of oxygen	↑	Arterial blood gas analysis	Mean difference (% of change); p-value Group A − 2.62 (3.50); 0.001 Group B − 9.25 (12.41); 0.001	Ali AA et al. [20]
partial pressure of carbon dioxide	↓	Arterial blood gas analysis	Mean difference (% of change); p-value Group A 1.64 (4.35); 0.001 Group B 3.95 (10.75); 0.001	Ali AA et al. [20]
Arterial blood pressure	↓	Sphygmomanometer	Mean difference (% of change); p-value Study group Systolic blood pressure −25.00 mmHg (16.67); < 0.001 Diastolic blood pressure −17.10 mmHg (18.08); < 0.001 Control group Systolic blood pressure −3.73 mmHg (2.48); 0.032 Diastolic blood pressure −0.90 mmHg (0.95); 0.01	Nagy EN et al. [39]
Serum lactate level	↓	Veinous blood sample	Mean difference (% of change); p-value Study group −0.82 mmol/L (51.57); < 0.001 control group −0.27 mmol/L (17.65); < 0.001	Nagy EN et al. [39]
Muscle mass	↔	Magnetic resonance imaging	MRI – mid arm in cm^2^ Group Mean ± SD in kg; p-value Baseline LAT 55.9 ± 1.7, HAT 56.3 ± 1.; 0.227 4 weeks LAT 57.5 ± 1.0, HAT 57.9 ± 0.9; 0.070 8 weeks LAT 58.9 ± 0.6, HAT 59.0 ± 0.5; 0.432 6 months LAT 61.4 ± 0.5, HAT 61.5 ± 0.2; 0.256	Nambi G et al. [19]
			MRI – mid thigh in cm^2^ Group Mean ± SD in kg; p-value Baseline LAT 63.4 ± 0.8, HAT 63.5 ± 0.8; 0.587 4 weeks LAT 65.3 ± 0.6, HAT 65.5 ± 0.6; 0.150 8 weeks LAT 68.4 ± 0.6, HAT 68.5 ± 0.6; 0.469 6 months LAT 72.5 ± 0.8, HAT 72.6 ± 0.8; 0.587	
			MRI - mid calf in cm^2^ Group Mean ± SD in kg; p-value Baseline LAT 60.2 ± 1.1, HAT 60.2 ± 1.1; 1.000 4 weeks LAT 65.2 ± 0.6, HAT 65.2 ± 0.6; 1.000 8 weeks LAT 66.3 ± 0.5, HAT 66.4 ± 0.5; 0.386 6 months LAT 68.7 ± 0.5, HAT 68.7 ± 0.5; 1.000	
Kinesiophobia	↓	Tampa scale of kinesiophobia	Group Mean ± SD; p-value Baseline LAT 32.1 ± 1.0, HAT 32.3 ± 0.9; 0.362 4 weeks LAT 23.5 ± 0.9, HAT 29.9 ± 0.9; 0.001 8 weeks LAT 18.0 ± 0.9, HAT 24.5 ± 1.4; 0.001 6 months LAT 13.5 ± 1.0, HAT 18.2 ± 1.0; 0.001	Nambi G et al. [19]
Risk of falls	↑	Timed-Up and Go Test	From 9.0 s to 7.3 s	Mayer KP et al. [21]
Functional status	↑	Short Performance Physical Battery	Total from 11 to 12	Mayer KP et al. [21]
Quality of life	↑	Sarcopenia and Quality of Life questionnaire	Group Mean ± SD; p-value Baseline LAT 57.3 ± 1.0, HAT 57.7 ± 1.0; 0.085 4 weeks LAT 63.0 ± 0.7, HAT 58.8 ± 0.9; 0.001 8 weeks LAT 69.0 ± 1.0, HAT 60.5 ± 0.8; 0.001 6 months LAT 72.6 ± 1.0, HAT 62.2 ± 0.8; 0.001	Nambi G et al. [19]
	↓	Health-related Quality of Life Questionnaire (Eq-5D-5 L)	Eq-5D-5 L (VAS 0–100): 50 to 40 Eq-5D-5 L (mean): 2.6 to 2.8	Mayer KP et al. [21]
Cognitive function	↑	Montreal Cognitive Assessment	27 to 28 (out of 30)	Mayer KP et al. [21]
	↔	Time-up and Go Test with cognitive component	From −34% to − 21% difference	
Distress	↑	Impact of Events Scale-Revised	Total: from 36 to 46 Mean response: from 1.6 to 2.1 Intrusion mean: from 1.6 to 1.9 Avoidance mean: from 0.75 to 1.25 Hyperarousal mean: from 2.8 to 3.5	Mayer KP et al. [21]
	↓	Perceived Stress Scale	Group Mean ± SD; p-value VR group from 23.1 ± 4.2 to 20.7 ± 3.5; 0.015 Control group from 23.8 ± 3.1. to 22.2 ± 3.8; 0.004	Rutkowski et al. [40]
Lung function	↔	Spirometry (expiratory volume for 1 s (FEV_1_); forced vital capacity (FVC); forced expiratory volume in one second of vital capacity (FEV_1_/VC))	Group Mean ± SD (mean); p-value FEV_1_ in l VR group from 2.8 ± 0.66 to 2.77 ± 0.68; 0.708 Control group from 2.72 ± 0.60 to 2.76 ± 0.61; 0.879 FVC in l VR group from 3.46 ± 0.78 to 3.56 ± 0.8; 0.087 Control group from 3.49 ± 0.86 to 3.5 ± 0.85; 0.168 FEV_1_/VC in % VR group from 84.3 (6.89) to 81.4 (7.77); 0.028 Control group from 82.4 (5.32) to 83.2 (10.1); 0.130	Rutkowski et al. [40]
		Plethysmography (total lung capacity (TLC))	Group Mean ± SD in l; p-value VR group from 5.18 ± 1.2 to 5.39 ± 1.1; 0.288 Control group from 4.9 ± 1.2 to 5.27 ± 1.0; 0.070	
Dyspnoea	↓	Modified Medical Research Council	Mean difference; p-value Study group 48.89%; *p* < 0.001 control group 12.81%; 0.001	Nagy EN et al. [39]
			From 2 to 1	Mayer KP et al. [21]
		Borg scale while 6-minute walk test	Group Mean (median); p-value VR group from 2.0 (3.0) to 0.0 (2.0); 0.033 Control group from 2.0 (2.75) to 0.5 (2.0); 0.004	Rutkowski et al. [40]
Muscle strength	↑	Medical Research Council sum score	From 55 to 59	Mayer KP et al. [21]
	↑	Lower extremity unilateral leg press	2-lb resistance (Watts): from 14.3 to 17.7 10% of body weight resistance (Watts): from 88 to 134	
	↑↓	Handgrip dynamometer	Right: from 24 kg to 33 kg Left: from 36 kg to 34 kg	Mayer KP et al. [21]
	↑		Group Mean ± SD in kg; p-value Baseline LAT 28.4 ± 0.7, HAT 28.5 ± 0.6; 0.505 4 weeks LAT 29.4 ± 0.5, HAT 29.2 ± 0.6; 0.118 8 weeks LAT 31.5 ± 0.6, HAT 29.8 ± 0.5; 0.001 6 months LAT 34.3 ± 0.8, HAT 30.4 ± 0.8; 0.003	Nambi G et al. [19]
Fatigue	↓	N. a.	N. a.	Santos S et al. [24]
Muscle strength	↑	Daniels Scale	From 2 to 4 out of 5	Santos S et al. [24]
Pain reduction	↓	Numeric Pain Scale	From 10 to 3 out of 10	Santos S et al. [24]
Range of motion	↑	Goniometer	Shoulder movements in ° Right pre/post physiotherapy; Left pre/post physiotherapy Flexion 100/160; 120/160 Extension 20/40; 30/40 Abduction 90/150; 120/160 Adduction 10/20; 30/30 External rotation 60/80; 80/80 Internal rotation 50/55; 60/60 Elbow movements in ° Right pre/post physiotherapy; Left pre/post physiotherapy Flexion 120/130; 130/140 Extension 0/0; 0/0	Santos S et al. [24]
Balance	↑	Unipodal Station Test	From 3 s to 8 s	Santos S et al. [24]
Basic activities of daily living	↑	N. a.	N. a.	Santos S et al. [24]
Instrumental activities of daily living	↑	N. a.	N. a.	Santos S et al. [24]
**Legend**	↑ increasing ↔ unchanged ↓ decreased			

#### Were adverse events reported for physiotherapeutic interventions in patients with post- or long- COVID syndrome?

In one case report the quality of life decreased [21]. But in most cases patients felt better regarding the quality of life [19, 20, 24, 39, 40].

#### Were negative influences reported for parallel, applied physiotherapeutic interventions in patients with post- or long-COVID syndrome?

Due to a lack of information, it is not possible to make a statement on this issue.

#### Were differences reported inpatient and out patient interventions?

Table 9 summarizes the identified practiced interventions and treatment parameters as the general frequency in mean. Only the 6 studies focusing on the subtype post-COVID-19 were considered because they provided enough information.

**Table 9:** Summarization on identified practised therapy interventions, their parameters and general frequency in mean for post-COVID-19

Healthcare delivery setting	Therapy category	Intervention	Treatment parameters	General frequency (in mean)	Study
Inpatient and Outpatient	Respiratory therapy	Diaphragma release Breathing exercises		3 sessions per week for 7 weeks	Ali AA et al. [20] Nagy EN et al. [39] Rutkowski et al. [40] Mayer KP et al. [21]
Inpatient and Outpatient	Aerobic training	Treadmill; Cycle ergometer	3–20 minutes 40–60% of maximum heart rate	3 sessions per week for 9 weeks	Ali AA et al. [20] Nambi G et al. [19] Mayer KP et al. [21]
Inpatient and Outpatient	Strength training	Free exercises, weights or weight machines	3 sets of 8–20 repititions, 1 minute rest	3 sessions per week for 9 weeks	Ali AA et al. [20] Nambi G et al. [19] Mayer KP et al. [21]

#### Critical appraisal

The studies selected for inclusion were subjected to critical appraisal by two independent researchers with extensive experience in the field of rehabilitation research, utilizing the JBI critical appraisal tools [37, 43]. The quality assessment was conducted to evaluate the methodological quality of the data to be extracted from the included articles. No manuscript was excluded during the critical appraisal process. The overall quality of the included literature was determined to be moderate. An overview of the critical appraisal is provided in Table 10.

**Table 11:** Overview on critical appraisal of the case reports following JBI critical appraisal tools [37]

	Item	**Mayer et al.** [21]	**Santos et al.** [24]
1	Patient characteristics clearly described	y	y
2	Patient history clearly described	y	y
3	Current clinical condition clearly described	y	y
4	Diagnostic tests/assessment methods clearly described	y	y
5	Intervention clearly described	y	n
6	Post-interventional clinical condition clearly described	y	y
7	Adverse/unanticipated events identified and described	y	n
8	Take-away lessons provided	y	y
	Total	8	6
Legend		y = yes n = no uc = unclear

**Table 10:** Overview on critical appraisal of the randomized controlled trials following JBI critical appraisal tools [43]

	Domain	Selection and allocation			Administration of intervention/exposure			Assessment, detection, and measurement of the outcome			Participant retention	Statistical conclusion validity		
Author	Question no.	1	2	3	4	5	6	7	8	9	10	11	12	13
Nagy et al. [39]	Dyspnoea: Modified Medical Research Council	y	y	y	y	n	y	y	y	y	y	y	y	y
	Fatigue: Fatigue Severity Scale										y	y	y	
	Walking distance: 6-minute walk test										y	y	y	
	Arterial blood pressure Sphygmomanometer										y	y	y	
	Serum lactate level: Veinous blood sample										y	y	y	
Nambi et al. [19]	Kinesiophobie: Tampa scale of kinesiophobia	y	y	y	y	y	y	y	y	y	y	y	y	y
	Muscle mass: Magnetic resonance imaging										y	y	y	
	Muscle strength: Handgrip dynamometer										y	y	y	
	Quality of life: Sarcopenia and Quality of Life questionnaire										y	y	y	
Rutkowski et al. [40]	Fatigue: Borg scale while 6-minute walk test	y	y	n	n	n	n	uc	y	y	y	y	y	y
	Walking distance: 6-minute walk test										y	y	y	
	Distress: Perceived Stress Scale										y	y	y	
	Lung function: a) Spirometry b) Plethysmography										a) y b) y	a) y b) y	a) y b) y	
	Dyspnoe: Borg scale while 6-minute walk test										y	y	y	
Ali et al. [20]	Fatigue: Fatigue Assessment Scale	y	uc	y	uc	uc	n	uc	y	y	n	y	y	y
	Walking Distance: 6-minute walk test										n	y	y	
	Arterial oxygen saturation: Arterial blood gas analysis										n	y	y	
	Partial pressure of oxygen: Arterial blood gas analysis										n	y	y	
	Partial pressure of carbon dioxide: Arterial blood gas analysis										n	y	y	
Total		4	3	3	2	1	1	2	4	4	12	17	17	4
Legend	y = yes n = no uc = unclear													

## Discussion

This scoping review aimed to give an overview on physiotherapeutic interventions and treatment parameters published in the scientific literature. The number of identified studies shows a gap in research on the topic which is contradictory to the recommendations of physiotherapy in the treatment of post- and long-COVID condition and the high number of prescribed physiotherapeutic remedies in the treatment of post- and long-COVID-19.

The included literature referring to physiotherapy interventions focus on respiratory therapy, aerobic and strength training. This scoping review reveals that the different interventions and techniques of respiratory therapy, aerobic and strength training were combined in each separate session. The majority of studies employed a set of three, with 10 to 15 repetitions and a one-minute rest interval between sets.

Only one of the included studies documented the practice of traditional physiotherapy techniques, including transcutaneous electrical nerve stimulation, friction massage and manual therapy [21]. This finding points out a research gap concerning the diversity of physiotherapeutic interventions in the treatment of patients with post-and long-COVID-19.

The assessments used in the studies were standardized assessments. Some of them do not meet the profile of physiotherapy, like spirometry or blood serum analysis. Others are highly practicable in the setting of outpatient physiotherapy, like the Borg scale or the 6-minute walk test. The included studies indicate reduction in fatigue, enhancements in respiratory function, and improvements in physical function. The quality of life increased in most cases, with the exception of one patient in one case report [21]. Pain is only assessed in one case report [24]. This finding raises the question whether pain reduction is a relevant outcome for post- and long-COVID-19 patients.

Medical guidelines and recommendations emphasize the treatment of physiotherapy [9, 44–46]. Several studies and reviews have demonstrated the positive impact of multidisciplinary rehabilitation programmes, even in infectious diseases [47, 48]. The post- and long-COVID-19 physiotherapy is based on the treatment of symptoms associated with certain diseases such as pain in fibromyalgia, cancer related fatigue or dyspnea in chronic obstructive pulmonary disease [10, 15]. Exercise programmes have been evaluated in post- and long-COVID research [18–21]. Commonly exercise programmes are carried out by sports and physiotherapists. A ‘slow progressive approach’ [18] or ‘pacing’ [44] is required in the treatment implying that therapeutic interventions must be introduced gradually and cautiously to promote adaption and minimize the risk of adverse reaction or symptom exacerbation [18, 28]. This therapeutic approach is demonstrated in the included case reports as they provide concrete insights to the physiotherapy treatment in time course [21, 24]. Further studies focusing on gradual adaption of therapeutic interventions are needed as they are recommended for practice [45].

The definitions of post- and long-COVID syndrome vary slightly with respect to the onset of symptoms following an acute infection [49]. Authors refer to the work of other authors or focus on describing the complex symptoms of this condition. This circumstance appears to influence the synthesis of physiotherapeutic interventions as the treatments provided were not aligned with the ICD-10 diagnosis.

Study limitations can be identified on the search strategy as no professional librarian supported the literature search. The involvement of a librarian could have improved the methodological quality of the literature search. as the literature research was limited to studies German and English published studies it may have led to the exclusion of relevant studies in other languages. Furthermore, grey literature was not considered, as it is only partially accessible and not published in a standardized manner. To ensure transparency and reproducibility the literature search was limited to peer-reviewed sources from established academic databases.

The number of identified studies publish until February 2023 addressing physiotherapy in post- and long-COVID-19 is very limited which also influences the statistical reporting. Study designs, outcomes and involved patient characteristics are very heterogenous making a direct comparison and quantitative synthesis difficult. The data was summarized descriptively which inhibits to draw conclusions about effectiveness of physiotherapy treatment in post- and long-COVID patients. Furthermore, treatment interventions were not described adequately detailed. For those reasons case reports were also included in the search beneath the low level of evidence, as they provide detailed insights on common therapeutic interventions applied from session to session.

However, from the findings of this scoping review objectives for further research can be derived as the gap in research is evident. Physiotherapy interventions addressing symptoms need to be evaluated with a higher methodological design and a higher sample size. A concrete definition of the included post- or long-COVID-19 diagnosis to specify the indication of physiotherapy is mandatory. Therefore, the ICD-10 codes could be useful. Nevertheless, influencing characteristics, especially in case reports, should be considered in health research as they provide a holistic picture of the participants. Therefore, the International Classification of Functioning, Disability and Health (ICF) contextual factors are appropriate [50]. Collecting these variables allows a more comprehensive understanding of participant profiles and the differentiation of treatment effects. These findings may contribute the development of clinical prediction rules by generating relevant predictors associated with treatment response within the specific clinical context.

Only a limited number of the identified publications provide detailed information regarding the specific treatment modalities practiced. The treatment methods and parameters vary across studies. For reproducibility, further research and translation into the health system it is necessary to provide detailed descriptions of the processes and the applied interventions. For example, there is a need to distinguish between a passive mobilization of the spine in general or a mobilization of a specific vertebral segment or a manipulation of a specific vertebral segment. Functional exercises need to be described more detailed additionally with pictures or videos. Standardized assessments need to be validated in the context of post- and long-COVID-19.

As physiotherapy comprises a broader spectrum of interventions beyond respiratory therapy, aerobic exercise, and strength training there are further physiotherapeutic approaches that need to be evaluated in pilot studies and randomized controlled trials. Future research should examine the efficacy of interventions such as manual therapy, passive mobilization, massage techniques, lymphatic drainage, relaxation therapies and neuromuscular treatment strategies in the context of different COVID-19 subtypes. Educational material used for training the therapists and assessors in the study also need to be provided in the publications for reproducibility and in particular to ensure the translation into practice.

## Conclusions

This scoping review highlights a high demand for research in the treatment of post- and long-COVID patients for the in-person physiotherapy setting. To ensure reproducibility and clinical applicability, a detailed description of all practiced physiotherapy techniques is essential. However, the results of this scoping review provide physiotherapists treatment options for patients with post- and long-COVID-19 as physiotherapy demonstrated to have positive trends in supporting health outcomes among patients with post- and long-COVID condition.

## Electronic supplementary material

Below is the link to the electronic supplementary material.

Supplementary Material 1

## Data Availability

All data used and/or analysed during the current study are available from the corresponding author on the reasonable request.
