# Supplementary material for: Physiotherapy interventions in post- and long-COVID-19: a scoping review of the literature up to February 2023
Source: BMC Health Serv Res. 2025 Oct 30;25:1425. doi: 10.1186/s12913-025-13631-7 (PMC12577318; doi:10.1186/s12913-025-13631-7)
Supplement: Supplementary file 1 — Supplementary Material 1 [file 12913_2025_13631_MOESM1_ESM.docx]

Appendix I: Search strategy

Web of Science

("Long COVID" OR "Post COVID-19" OR "Post-acute COVID") AND ("physiotherapy" OR "aerobic exercise" OR "physical activity" OR "passive mobilization" OR "active mobilization" OR "manual therapy" OR "massage therapy" OR "lymphatic drainage" OR "electrotherapy" OR "ultrasound" OR "respiratory therapy" OR "Proprioceptive neuromuscular Facilitation" OR "Vojta" OR "Bobath" OR "pressure ulcer management" OR "prevention") AND ("Physical fitness" OR "fatigue" OR "general health" OR "pain" OR "return to work" OR "physical health" OR "mental health" OR "mobility" OR "vital capacity" OR "fatigue" OR "dyspnoe" OR "myalgia" OR "arthralgia" OR "functional capacity" OR "health related quality of life" OR "stress" OR "range of movement" OR "Patient reported outcome measures") AND ("Randomized control trial" OR "Observational studies " OR "Case reports" OR "Systematic reviews" OR "Meta-analysis") NOT ("study protocol" OR "telemedicine") (Topic)

("Long COVID") AND ("physiotherapy")

CINAHL

TI ( ("Long COVID" OR "Post COVID-19" OR "Post-acute COVID") AND ("physiotherapy" OR "aerobic exercise" OR "physical activity" OR "passive mobilization" OR "active mobilization" OR "manual therapy" OR "massage therapy" OR "lymphatic drainage"OR "electrotherapy" OR "ultrasound"OR "respiratory therapy" OR "Proprioceptive neuromuscular Facilitation" OR "Vojta" OR "Bobath"OR "pressure ulcer management" OR "prevention") AND (OR "Physical fitness" OR "fatigue"OR "general health" OR "pain"OR "return to work" OR "physical health"OR "mental health" OR "mobility"OR "vital capacity" OR "fatigue"OR "dyspnoe" OR "myalgia"OR "arthralgia" OR "functional capacity" OR "health related quality of life" OR "stress" OR "range of movement" OR "Patient reported outcome measures") AND (OR "Randomized control trial" OR "Observational studies " OR "Case reports" OR "Systematic reviews" OR "Meta-analysis") NOT ("study protocol" OR "telemedicine"))

TI (("Long COVID" OR "Post-acute COVID-19" OR "Post-acute COVID") AND ("physiotherapy" OR "aerobic exercise" OR "passive mobilization" OR "active mobilization" OR "manual therapy" OR "massage therapy" OR "lymphatic drainage"OR "electrotherapy" OR "ultrasound"OR "respiratory therapy" OR "Proprioceptive neuromuscular Facilitation" OR "Vojta" OR "Bobath"OR "pressure ulcer management" OR "prevention") AND (OR "Physical fitness" OR "fatigue"OR "general health" OR "pain"OR "return to work" OR "physical health"OR "mental health" OR "mobility"OR "vital capacity" OR "fatigue"OR "dyspnoe" OR "myalgia"OR "arthralgia" OR "functional capacity" OR "health related quality of life" OR "stress" OR "range of movement" OR "Patient reported outcome measures") AND (OR "Randomized control trial" OR "Observational studies " OR "Case reports" OR "Systematic reviews" OR "Meta-analysis") NOT ("study protocol" OR "telemedicine"))

TI (("Long COVID" OR "Post-acute COVID") AND ("physiotherapy" OR "aerobic exercise" OR "passive mobilization" OR "active mobilization" OR "manual therapy" OR "massage therapy" OR "lymphatic drainage"OR "electrotherapy" OR "ultrasound"OR "respiratory therapy" OR "Proprioceptive neuromuscular Facilitation" OR "Vojta" OR "Bobath"OR "pressure ulcer management" OR "prevention") AND (OR "Physical fitness" OR "fatigue"OR "general health" OR "pain"OR "return to work" OR "physical health"OR "mental health" OR "mobility"OR "vital capacity" OR "fatigue"OR "dyspnoe" OR "myalgia"OR "arthralgia" OR "functional capacity" OR "health related quality of life" OR "stress" OR "range of movement" OR "Patient reported outcome measures") AND (OR "Randomized control trial" OR "Observational studies " OR "Case reports" OR "Systematic reviews" OR "Meta-analysis") NOT ("study protocol" OR "telemedicine"))

PEDro

"Long COVID" AND "Physiotherapy"

"Long COVID" AND "physical therapy"

Post COVID-19

Post-acute COVID

"Post-acute COVID" AND "Physiotherapy"

"Post-acute COVID" AND "physical therapy"

"Post-acute COVID" AND "aerobic excersise"

"Post-acute COVID" AND "excersise"
